# Ozone stress-induced DNA methylation variations and their transgenerational inheritance in foxtail millet

**DOI:** 10.3389/fpls.2024.1463584

**Published:** 2024-09-25

**Authors:** Long Wang, Yang Liu, Xiaohan Song, Shiji Wang, Meichun Zhang, Jiayi Lu, Sheng Xu, Hongyan Wang

**Affiliations:** ^1^ Laboratory of Plant Epigenetics and Evolution, School of Life Sciences, Liaoning University, Shenyang, China; ^2^ Academy of Agricultural and Forestry Sciences, Qinghai University, Xining, China; ^3^ Institute of Broomcorn Millet, Zhangjiakou Academy of Agricultural Sciences, Zhangjiakou, China; ^4^ Chinese Academy of Sciences (CAS) Key Laboratory of Forest Ecology and Management, Institute of Applied Ecology, Shenyang, China

**Keywords:** ozone, DNA methylation, transgenerational inheritance, MSAP (methylation sensitive amplified polymorphism), foxtail millet (*Setaria italica* L.)

## Abstract

Elevated near-surface ozone (O_3_) concentrations have surpassed the tolerance limits of plants, significantly impacting crop growth and yield. To mitigate ozone pollution, plants must evolve a rapid and effective defense mechanism to alleviate ozone-induced damage. DNA methylation, as one of the most crucial epigenetic modifications, plays a pivotal role in maintaining gene stability, regulating gene expression, and enhancing plant resilience to environmental stressors. However, the epigenetic response of plants to O_3_ stress, particularly DNA methylation variations and their intergenerational transmission, remains poorly understood. This study aims to explore the epigenetic mechanisms underlying plant responses to ozone stress across generations and to identify potential epigenetic modification sites or genes crucial in response to ozone stress. Using Open Top Chambers (OTCs), we simulated ozone conditions and subjected foxtail millet to continuous ozone stress at 200 nmol mol^-1^ for two consecutive generations (S0 and S1). Results revealed that under high-concentration ozone stress, foxtail millet leaves exhibited symptoms ranging from yellowing and curling to desiccation, but the damage in the S1 generation was not more severe than that in the S0 generation. Methylation Sensitive Amplified Polymorphism (MSAP) analysis of the two generations indicated that ozone stress-induced methylation variations ranging from 10.82% to 13.59%, with demethylation events ranged from 0.52% to 5.58%, while hypermethylation occurred between 0.35% and 2.76%. Reproductive growth stages were more sensitive to ozone than vegetative stages. Notably, the S1 generation exhibited widespread demethylation variations, primarily at CNG sites, compared to S0 under similar stress conditions. The inheritance pattern between S0 and S1 generations was mainly of the A-A-B-A type. By recovering and sequencing methylation variant bands, we identified six stress-related differential amplification sequences, implicating these variants in various biological processes. These findings underscore the potential significance of DNA methylation variations as a critical mechanism in plants’ response to ozone stress, providing theoretical insights and references for a comprehensive understanding of plant adaptation mechanisms to ozone stress and the epigenetic role of DNA methylation in abiotic stress regulation.

## 1 Introduction

Ozone (O_3_) is a colorless gas with a pungent odor and strong oxidative properties, playing a crucial role in both the troposphere and stratosphere (Conte et al., 2021; Wedow et al., 2021). With industrial development and the extensive use of fossil fuels and nitrogen-containing fertilizers, nitrogen oxides and volatile organic compounds accumulate in the atmosphere, leading to a continuous rise in ozone concentrations in the ground-level atmosphere (Prather et al., 2003; The Royal Society, 2008). Recent studies indicate that near-surface ozone concentrations have exceeded the tolerance limits of plants (Ashmore, 2005). High concentrations of ozone can enter plant tissues through leaf stomata, generating reactive oxygen species (ROS) such as hydrogen peroxide and hydroxyl radicals through oxidative decomposition. These ROS adversely affect plants by reducing chlorophyll content, inhibiting carbohydrate synthesis, and compromising antioxidant enzyme activities (Hasan et al., 2021; Li et al., 2024). This oxidative stress causes severe toxic damage to plants, resulting in decreased photosynthetic efficiency, weakened resilience to (a)biotic stressors, and potential yield reductions (Begum et al., 2020).

Research indicates that China’s average ozone concentration of 30.9 ppm h, compared to 17.5 ppm h in Japan and 21.2 ppm h in South Korea, has led to wheat and rice yield losses exceeding 20%, causing significant economic damage (Feng et al., 2022). It is projected that by 2030, ozone pollution could further decrease global wheat production by 5.4% to 26%, soybean by 15% to 19%, and maize by 4.4% to 8.7% (Sinha et al., 2015). Due to their sessile nature, plants must respond appropriately to adverse environmental stresses (Hayes et al., 2021; Praat et al., 2021). Epigenetic mechanisms play a pivotal role in shaping stress responses and establishing stress memory in plants (Bourguet et al., 2021; Perrella et al., 2022; Kumar and Rani, 2023). Studies indicate that stress memory, regulated by DNA methylation, can faithfully transmit stress-induced epigenetic variations to subsequent generations, thereby facilitating plant evolution and enhancing environmental adaptability (Mauch-Mani et al., 2017; López Sánchez et al., 2021; Sun et al., 2022). For instance, previous research utilizing Methylation Sensitive Amplified Polymorphism (MSAP) analysis on rice subjected to salt and alkali stresses revealed alterations in DNA methylation patterns. Subsequent analyses of offspring (S1 and S2 generations) demonstrated the persistence of these methylation patterns and their associated stress tolerance (Feng et al., 2012). Similarly, investigations on rice under low nitrogen stress showed inheritable changes in DNA methylation patterns in the S0 generation. When subjected to the same stress, the progeny inherited these patterns and exhibited tolerance to low nitrogen stress (Kou et al., 2011). Moreover, studies on Boechera stricta of the Brassicaceae family under drought stress found that offspring displayed enhanced drought tolerance, accompanied by reduced DNA methylation levels and decreased secondary metabolite, glucosinolate (Alsdurf et al., 2015). Research on rice under heavy metal stress observed upregulated expression of stress-responsive genes (HMAs), followed by three generations of methylation pattern analysis, revealing transgenerational inheritance of altered DNA methylation patterns under heavy metal stress (Cong et al., 2019). These findings underscore the association between changes in plant DNA methylation levels and patterns and their responses to abiotic stressors, highlighting the faithful transmission of stress-induced DNA methylation variations to offspring.

Research suggests that as ozone levels continue to rise due to industrialization, it has become a primary abiotic stressor threatening crop growth and yields (Gupta et al., 2022, 2023; Singh et al., 2023). Foxtail millet (*Setaria italica.* L), an ancient and important cereal crop in East Asia known for its drought, salt, and nutrient-poor soil tolerance (Nadeem et al., 2020; He et al., 2024). Therefore, foxtail millet is considered an ideal model for global climate change research due to its valuable traits, including worldwide distribution, tolerance to various conditions, a compact genome, and a short growth period (He et al., 2023). Previous studies have demonstrated that ozone stress alters the DNA methylation patterns across the entire genome of plants (*Oryza sativa* L.) (Wang et al., 2023), but the variations in DNA methylation across different generations under ozone stress and their modes of transmission remain unclear. Therefore, this study utilizes MSAP technology to uncover the DNA methylation levels and patterns in foxtail millet under high-concentration ozone stress and explores the genetic regulations of DNA methylation variations across different generations. This research aims to provide insights into the resistance mechanisms of plants to ozone stress and the transgenerational epigenetic transmission, thereby contributing to the global food security and the resistance breeding of foxtail millet.

## 2 Materials and methods

### 2.1 Plant materials and O_3_ stress treatment

This study employed the foxtail millet variety Chaogu 58 provided by the Liaoning Institute of Dryland Agriculture and Forestry. Five hundred seeds were germinated in darkness at 25°C for three days. Germinated seedlings were then planted in growth chambers under a 28°C/22°C (light/dark) cycle with 60-70% humidity (Zhao et al., 2019). Upon full expansion of the third leaf, seedlings (S0 generation) were evenly transferred to Open Top Chambers (OTCs) with/without ozone stress. The treatment plants were grown in OTCs for ozone fumigation at a concentration of 200 nmol·mol-1. Control plants were grown in OTCs without an ozone generator, maintaining an air concentration of approximately 40 nmol·mol-1 as the baseline. Samples were collected on the 10th (S0-1-10d) and 30th (S0-1-30d) days of treatment. Subsequently, the millet plants were grown to maturity within the respective OTCs. Seeds collected in the following year constituted the S1 generation, and the S1 generation seedlings were subjected to the same ozone stress conditions. Before treatment and on the 30th day of treatment, ten individual plants derived from different S0 offsprings were randomly selected for sampling (S1-X-0d and S1-X-30d, respectively, where X represents individual plant numbers from 1 to 10). Each treatment had three biological replicates. The OTC system design followed principles outlined by Xu et al (Wang et al., 2022), ensuring controlled ozone exposure throughout the experiment. Leaf tissue samples collected at different treatment durations were flash-frozen in liquid nitrogen and stored at -80°C for DNA methylation analysis. The antepenultimate leaves were used to investigate the phenotypic damage of millet plants.

### 2.2 MSAP Analysis

Genomic DNA was extracted from the fourth to the sixth expanded leaves of Chaogu 58 using a modified CTAB method (Kidwell and Osborn, 1992). Subsequently, the Methylation-Sensitive Amplification Polymorphism (MSAP) technique was employed to comprehensively analyze the DNA methylation levels, patterns, and specific methylation sites across different generations of foxtail millet (Yaish et al., 2014; Yu et al., 2011). Detailed information regarding the amplification adapters and primers used in the experiment is provided in **Supplementary Table S1**. To accurately calculate DNA methylation levels, only loci that consistently appeared or were absent in all three replicates were analyzed. The following formula was used for analysis (MSAP%) (Wang et al., 2023):


MSAP(%)=[(II+III+IV)/(I+II+III+IV)]×100%

Type I indicates that the CCGG site is not methylated, Type II indicates that the CCGG site is hemi-methylated, Type III and Type IV indicates that the CCGG site is fully methylated.

### 2.3 Statistical methods for analyzing methylation variation inheritance across generations

To investigate whether ozone stress induces transgenerational transmission of methylation variations through meiosis and to discern the mechanisms of such transmission, we conducted analyses four types of samples across different generations, including the pre-ozone-treated S0 generation (CK-S0), the post-ozone-treated S0 generation (S0-X-30d), the pre-ozone-treated S1 generation (S1-X-0d), and the post-ozone-treated S1 generation (S1-X-30d). The comparison sequence was (CK-S0) - (S0-200-30d) - (S1-X-0d) - (S1-X-30d). Based on the methylation patterns of each sample that were statistically calculated in 2.2, the methylation variations among the four samples were determined. Instances where no change in methylation patterns occurred before and after ozone treatment were denoted by identical letters (e.g., A-A or B-B), while variations in methylation patterns were represented by different letters (e.g., A-B or A-B-C-D).

### 2.4 Cloning and sequencing of variant bands

Methylation variant bands were excised from the gels and incubated at 37°C for 8 hours. Subsequently, the supernatants were centrifuged and collected for re-amplification using a pre-amplification primer set and protocol. Further steps, including ligation and transformation, were performed using pUM19-T vector and DH-5α competent cells (Beijing Dingguo Changsheng Biotechnology Co., Ltd., Beijing, China). Recombinant DNA fragments were cloned and sequenced based on sequence characteristics.

### 2.5 DNA sequence alignment and homology search

BioEdit software was used to remove adapters and primers from DNA sequences. Candidate sequences were then queried and aligned using Phytozome (https://phytozome-next.jgi.doe.gov/) and NCBI (http://blast.ncbi.nlm.nih.gov/Blast.cgi), with Blast N and Blast X employed for sequence analysis and homology searches within the foxtail millet genome.

## 3 Results

### 3.1 Ozone stress induces direct physiological damage to plant leaves

**Figure 1:**
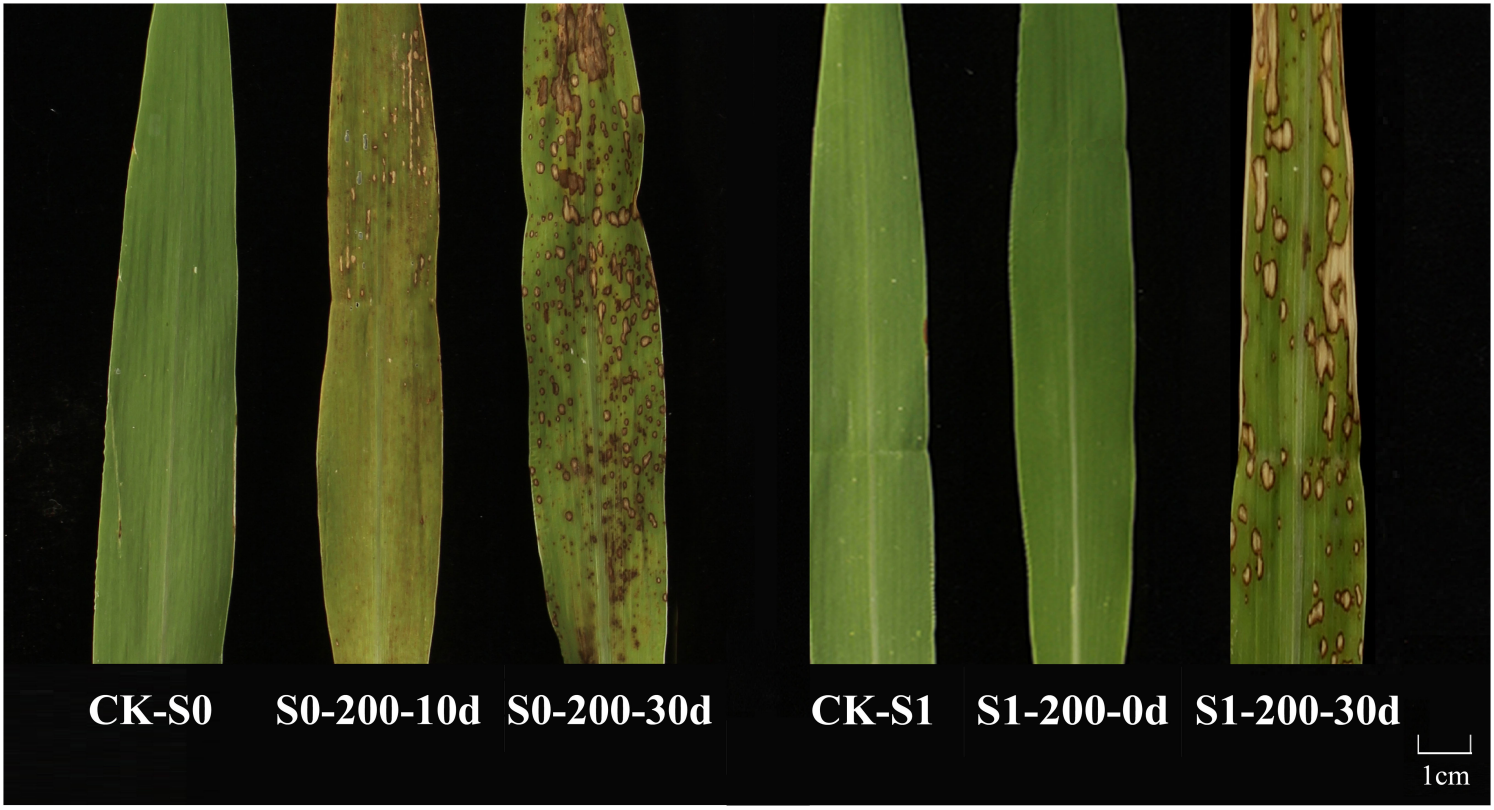
Phenotypic damage to foxtail millet leaves across different generations under ozone stress (200 nmol·mol^-1^). CK-S0: Control for S0 generation; S0-200-10d: Millet of the S0 generation treated with 200 nmol·mol^-1^ ozone concentration for 10 days; S0-200-30d: Millet of the S0 generation treated with 200 nmol·mol^-1^ ozone concentration for 30 days; CK-S1: Control for S1 generation; S1-200-0d: S1 generation millet untreated with ozone; S1-200-30d: S1 generation millet treated again with 200 nmol·mol^-1^ ozone concentration for 30 days.

The study revealed that ozone stress directly damages plant leaves, with significant effects (Ting and Mukerji, 1971). By subjecting successive generations (S0 and S1) of foxtail millet to ozone treatment (**Figure 1**), it was observed that compared to the control group, exposure to 200 nmol·mol^-1^ ozone concentration resulted in visible phenotypic damage to S0 generation millet leaves, worsening with longer treatment durations. Specifically, after 10 days of ozone exposure, S0 millet leaves exhibited curling and irregular light yellow necrotic spots; by the 30th day of treatment, severe wrinkling and extensive brown necrotic patches were evident. With successive generations, S1 millet under ozone stress for 30 days also displayed similar phenotypic damage. These findings indicate that 200 nmol·mol^-1^ ozone severely disrupts millet leaf growth, with damage increasing with prolonged exposure.

### 3.2 Ozone stress induces extensive methylation changes in the foxtail millet genome across different generations

**Figure 2:**
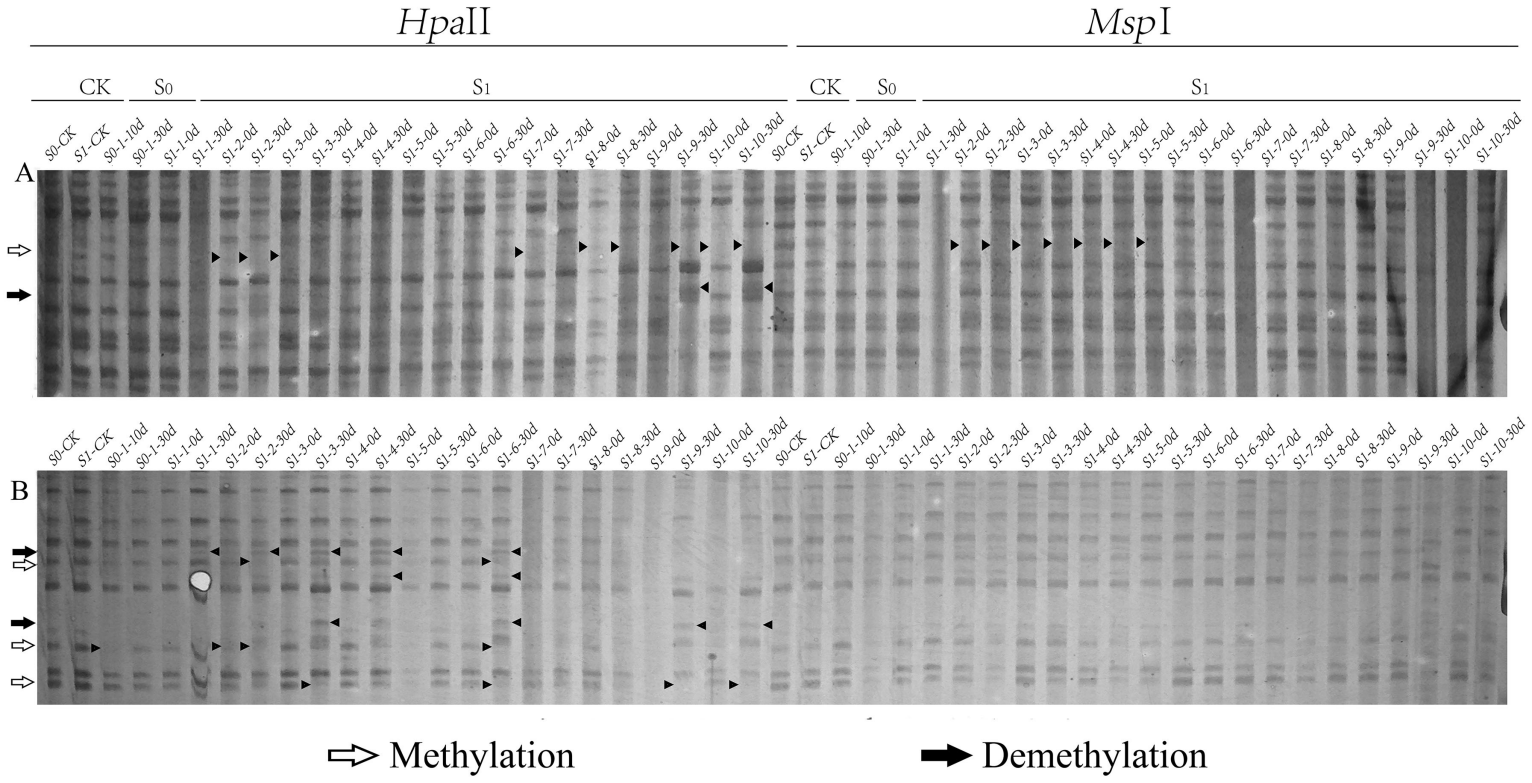
Differential patterns of DNA methylation variations in foxtail millet across different generations (S0 and S1) under ozone stress treatment. Primer combinations **(A)** E-AGA *H/M*-TCT, **(B)** E-ACT *H/M*-TTA, with specific methylation variation sites indicated by black triangles in the figure.

As analyzed using MSAP technology to assess methylation variations post ozone exposure (**Table 1**, **Figure 2**), a total of 1613-1683 clear methylation bands, representing the cumulative 1737 distinct methylation modification sites were amplified. Analysis of methylation levels revealed an overall reduction induced by ozone stress, albeit with variations among generations. Specifically, compared to S0-CK, ozone-treated S0 generation showed a slight decrease in overall genome methylation from 13.47% to 13.3%, indicating ozone stress induces subtle adjustments in foxtail millet’s genomic methylation. Following re-exposure of S1 generation to ozone, apart from a rise in methylation levels in the individual S1-10 (S1-10-30d) (13.59%), the remaining samples exhibited methylation levels lower than S1-CK, ranging from 11.17% to 13.41%. This indicates that continuous ozone treatment induces broader methylation variations in millet. Interestingly, randomly selected S1 plants, even without ozone exposure (labeled as S1-X-0d), showed lower overall genome methylation levels compared to controls (S1-CK), ranging from 10.82% to 13.24%. Upon ozone re-treatment, they displayed diverse DNA methylation alterations. However, the methylation levels of most plants increased, and they remained lower than the control, indicating that ozone stress indeed induces extensive methylation changes in foxtail millet across different generations, with varying plant responses to ozone stress at different growth stages, particularly heightened sensitivity during reproductive stages compared to nutritional growth phases.

**Table 1:** DNA methylation levels across different generations under ozone stress treatment.

Type	O_3_ Stress Time-Point																							
	CK		S0		S1																			
	S0– CK	S1– CK	S0–1 –10d	S0–1 –30d	S1–1 –0d	S1–1 –30d	S1–2 –0d	S1–2 –30d	S1–3 –0d	S1–3 –30d	S1–4 –0d	S1–4 –30d	S1–5 –0d	S1–5 –30d	S1–6 –0d	S1–6 –30d	S1–7 –0d	S1–7 –30d	S1–8 –0d	S1–8 –30d	S1–9 –0d	S1–9 –30d	S1–10 –0d	S1–10 –30d
I	1503	1503	1506	1506	1508	1509	1527	1514	1537	1507	1549	1513	1516	1543	1529	1504	1512	1504	1526	1515	1510	1508	1507	1501
II	43	43	40	40	60	39	66	52	62	40	69	41	37	65	79	40	62	42	71	42	77	53	83	44
III	69	69	69	69	64	68	62	69	63	67	65	72	79	61	63	69	70	73	68	78	84	65	78	77
IV	122	122	122	122	105	121	82	102	75	123	54	111	105	68	66	124	93	118	72	102	66	111	69	115
Total sites	1737	1737	1737	1737	1737	1737	1737	1737	1737	1737	1737	1737	1737	1737	1737	1737	1737	1737	1737	1737	1737	1737	1737	1737
Total amplified bands	1615	1615	1615	1615	1632	1616	1655	1635	1662	1614	1683	1626	1632	1669	1671	1613	1644	1619	1665	1635	1671	1626	1668	1622
Total methylated bands	234	234	231	231	229	228	210	223	200	230	188	224	221	194	208	233	225	233	211	222	227	229	230	236
MSAP (%) ^a^	13.47	13.47	13.30	13.30	13.18	13.13	12.09	12.84	11.51	13.24	10.82	12.90	12.72	11.17	11.97	13.41	12.95	13.41	12.15	12.78	13.07	13.18	13.24	13.59
Fully methylated bands	191	191	191	191	169	189	144	171	138	190	119	183	184	129	129	193	163	191	140	180	150	176	147	192
Fully methylated ratio (%)^b^	11.00	11.00	11.00	11.00	9.73	10.88	8.29	9.84	7.94	10.94	6.85	10.54	10.59	7.43	7.43	11.11	9.38	11.00	8.06	10.36	8.64	10.13	8.46	11.05
Hemi-methylated ratio(%)^c^	2.48	2.48	2.30	2.30	3.45	2.25	3.80	2.99	3.57	2.30	3.97	2.36	2.13	3.74	4.55	2.30	3.57	2.42	4.09	2.42	4.43	3.05	4.78	2.53
Non-methylated ratio (%) ^d^	86.53	86.53	86.70	86.70	86.82	86.87	87.91	87.16	88.49	86.76	89.18	87.10	87.28	88.83	88.03	86.59	87.05	86.59	87.85	87.22	86.93	86.82	86.76	86.41

a MSAP(%)=[(II+III+IV)/(I+II+III+IV)]×100%.

b Fullymethylatedratio(%)=[(III+IV)/(I+II+III+IV)]×100%.

c Hemi-methylatedratio(%)=[(II)/(I+II+III+IV)]×100%.

d Non-methylatedratio(%)=[(I)/(I+II+III+IV)]×100%.

**Figure 3:**
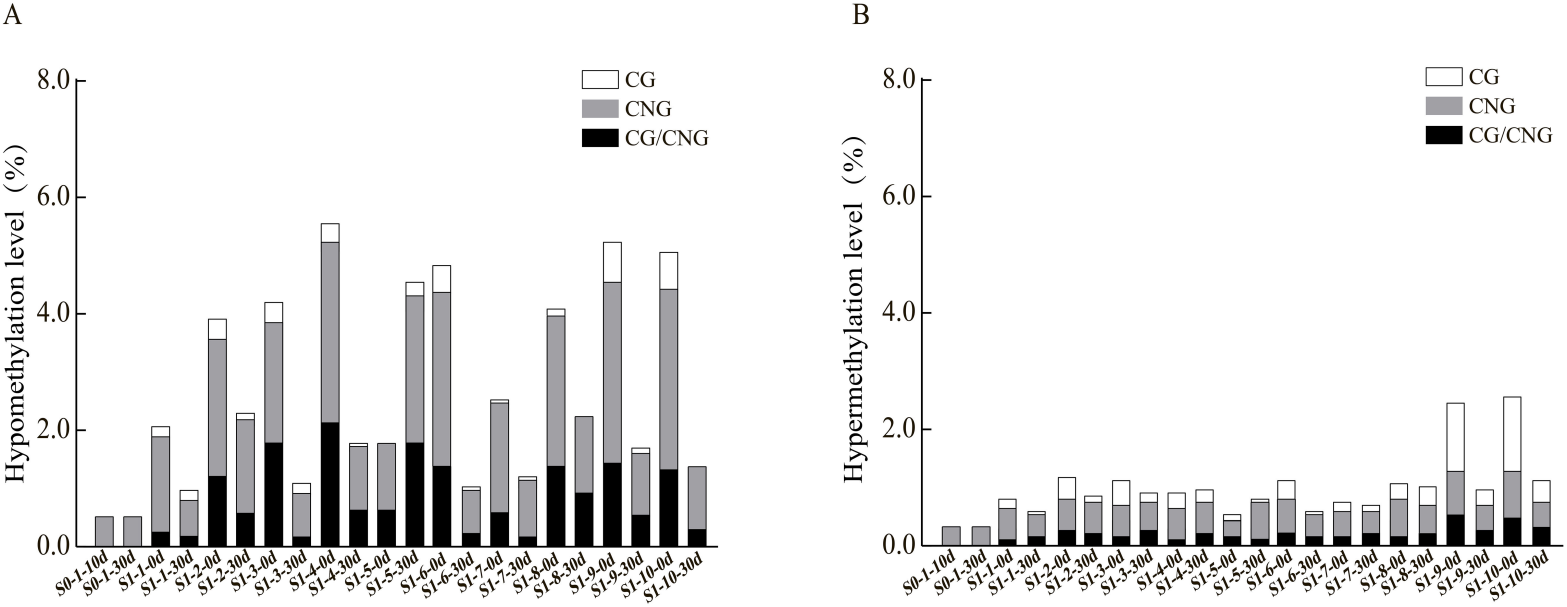
Analysis of hypomethylation and hypermethylation levels at different sites in foxtail millet across different generations (S0 and S1) under ozone stress treatment. **(A)** Hypomethylation at CG, CNG or CG/CNG sites, **(B)** Hypermethylation at CG, CNG or CG/CNG sites.

To further explore the patterns and characteristics of DNA methylation variations induced by ozone stress across different generations of foxtail millet, we analyzed the hypomethylation and hypermethylation variation sites in S0 and S1 generations (**Figure 3**). Our results indicate that demethylation variations in S0 primarily occurred at CNG sites (0.52%) (**Figure 3A**). After ozone re-stress, demethylation variations in S1 increased to 0.98% to 4.55%, predominantly at CNG sites (0.63% to 2.53%). Moreover, continuous ozone stress led to a gradual activation of demethylation changes at CG and CG/CNG sites in S1, with variation rates ranging from 0.06% to 0.23% and 0.17% to 1.79%, respectively. This suggests that demethylation variations increased with successive ozone stress generations. However, compared to the S1 generation plants not treated by ozone, which showed a demethylation range of 1.78% to 5.58%, the demethylation variations in S1 individuals exposed to ozone for 30 days (S1-X-30d) appear attenuated but still lower than the control.

Regarding hypermethylation patterns (**Figure 3B**), hypermethylation in S0 under ozone stress was 0.35%, while in S1, it ranged from 0.63% to 1.21%, predominantly both at CNG sites (0.40% to 0.69%). Additionally, the S1 showed new hypermethylation changes at CG (0.06%-0.40%) and CG/CNG (0.12%-0.35%) sites. Compared to treated S1 individuals, hypermethylation in untreated S1 ranged from 0.58% to 2.76% (CNG: 0.29% to 0.86%, CG: 0.12% to 1.38%, CG/CNG: 0.12% to 0.58%), with reductions mainly observed at CG sites. Overall, ozone stress induced extensive hypomethylation and hypermethylation variations in the foxtail millet genome, with more pronounced effects in S1, suggesting that this generation may regulate more functional gene expression in response to ozone stress through changes in methylation patterns.

### 3.3 Analysis of the inheritance modes of DNA methylation variations induced by ozone stress

**Figure 4:**
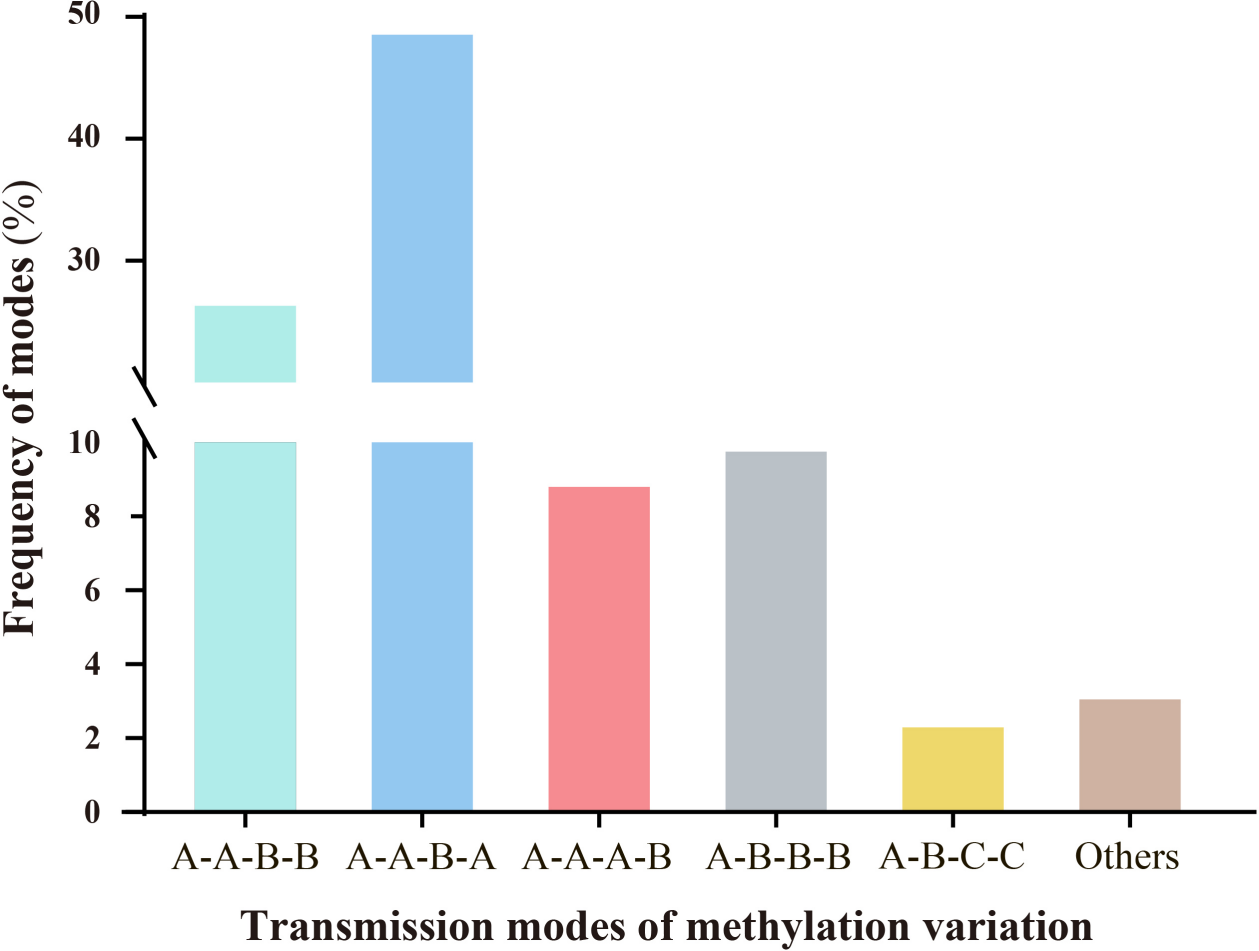
Analysis of the inheritance modes of DNA methylation variations in millet across different generations after ozone stress. The sample sequence is (CK-S0) - (S0-200-30d) - (S1-X-0d) - (S1-X-30d); letters represent the similarities and differences in DNA methylation patterns among the four samples. (1).A-A-B-B: There are no methylation variations in the S0 generation leaves, but variations arise during the reproductive phase under stress and are transmitted to the offspring. The S1 generation undergoes re-stress, the variation pattern remains unchanged. (2).A-A-B-A: The S1 generation undergoes re-stress, the variation pattern changes and reverts to the parental type. (3).A-A-A-B: Neither the S0 nor S1 generations exhibit methylation variations, but upon re-stress in the S1 generation, new mutations appear. (4).A-B-B-B: Methylation variations appear in the S0 generation and are stably transmitted to the offspring. Even after the offspring are stressed by ozone, they still maintain this mutation pattern. (5).A-B-C-C: The S0 generation leaf and reproductive phase show different variation patterns. Upon re-stress in the S1 generation, these variations remain stable.

Persistent environmental stress exerts plasticity on epigenetic modifications. Under stress, epigenetic variations, particularly DNA methylation changes, can be transmitted through mitosis and/or meiosis, playing significant roles in plant genome evolution and adaptation to adversity (Riddle and Richards, 2005; Bourguet et al., 2021). Therefore, we investigated whether ozone stress could transmit epigenetic variations across generations via meiosis and examined the modes of transmission. Results indicated that different modes of methylation variation transmission across generations could be categorized into six types, each with varying frequencies (**Figure 4**). These primarily included A-A-B-B (26.72%), A-A-B-A (48.73%), A-A-A-B (8.97%), A-B-B-B (9.92%), A-B-C-C (2.46%), and others (3.2%). The predominant types are characterized by scenarios where progeny of the S0 generation undergo stress-induced variations during reproductive growth, passing them on to subsequent generations with either unchanged (A-A-B-B) or altered patterns consistent with the parent (A-A-B-A). Additionally, three other types describe scenarios where no variation occurs in the S0 generation, but new variations emerge in subsequent generations following stress (A-A-A-B); where variations from the S0 generation persist through subsequent generations even after ozone stress (A-B-B-B); and where new methylation changes occur in the S0 generation under ozone stress, but nutritional and reproductive phases show different variation patterns. Upon re-stress in the S1 generation, these variations remain stable (A-B-C-C).

Overall, millet shows increased sensitivity to ozone stress during the reproductive growth stage, leading to more pronounced methylation variations. Moreover, most of the methylation variation sites (39.1%, which is the sum of A-A-B-B, A-B-B-B and A-B-C-C) produced in the S0 generation are likely to be maintained or further altered in the S1 generation.

### 3.4 Analysis of DNA methylation variation sequences in millet after ozone stress treatment

To investigate the sequence characteristics of DNA methylation mutations under ozone stress and to explore potential candidate genes responsive to ozone stress, we sequenced 202 mutated bands. We discovered that six sequences share homology with known millet gene sequences and are involved in various biological processes (**Table 2**). These include a biological rhythm regulation protein (TIME FOR COFFEE), an adenosine-binding protein, a membrane-associated calcium-binding protein, an ABC transporter, and a histone acetyltransferase. Among these, MS7, MS92, and MS178 belonged to the A-A-B-B type, while MS54, MS120, and MS192 were classified as A-A-B-A types, which indicate that these DNA methylation variation sequences may play a more significant role in millet’s response to ozone stress.

**Table 2:** Chromosome location and homology analysis of variant sequences.

Fragment	Chromosome	Methylation status under stress	Methylation variation transmission mode	Accession	Description	E-Value	Size (bp)
MS7	Chr.02	Demethylated	A-A-B-B	LOC101777931	Protein TIME FOR COFFEE	1 x 10^-5^	153
MS54	Chr.06	Demethylated	A-A-B-A	LOC101782192	Polyadenylate-binding protein RBP45-like	4 x 10^-22^	247
MS92	Chr.01	Demethylated	A-A-B-B	LOC101777330	Plasma membrane-associated cation-binding protein	8 x 10^-69^	216
MS120	Chr.09	Demethylated	A-A-B-A	LOC101783503	ABC transporter G family member 11-like	2 x 10^-17^	109
MS178	Chr.06	Demethylated	A-A-B-B	LOC101782192	Polyadenylate-binding protein RBP45-like	1 x 10^-22^	249
MS192	Chr.09	Demethylated	A-A-B-A	XP_004958388.1	MYST-like histone acetyltransferase 1	1 x 10^-2^	241

## 4 Discussion

Research have shown that abiotic stress can induce changes in whole-genome cytosine methylation in plants, and these stress-induced DNA methylation variations can be stably transmitted to offspring through mitosis and meiosis (Mauch-Mani et al., 2017; López Sánchez et al., 2021; Sun et al., 2022). Additionally, plants can regulate the expression of stress response genes through dynamic changes in whole-genome cytosine methylation, thereby conferring stress tolerance (Kou et al., 2011; Feng et al., 2012). Therefore, we analyzed the DNA methylation levels and patterns in millet under ozone stress using MSAP technology and explored the hereditary patterns of DNA methylation variations between different generations. The results indicate that under ozone stress (200nmol·mol^-1^), both the S0 and S1 generations of millet exhibited visible phenotypic damage and necrotic lesions, with the severity of damage increasing over the duration of the exposure. This may be due to ozone penetrating the plant tissue, reacting with internal substances to form reactive oxygen species (ROS), which accelerates cellular aging in the leaves, causing localized necrosis and eventually leading to desiccation (**Figure 1**) (Nowroz et al., 2024). Studies have shown that methylation changes in plant play a significant role in the response to various abiotic stresses (adaptation), where important resistance genes (such as those responding to drought, salt, and cold stresses) can be re-expressed or expressed at higher levels through demethylation when plants are stressed, enhancing their ability to withstand adverse conditions (Pandey et al., 2017; Fan et al., 2024; Naderi et al., 2024). In this study, MSAP analysis revealed that the millet genome in different generations mainly showed changes in full methylation and hemimethylation levels, and compared to the control group, the DNA methylation level in the S0 generation under ozone treatment decreased (0.17%). Similar results were reported in previous studies on rice. Demethylation was the primary type of methylation change at three different concentrations of ozone stresses in rice. Moreover, with the extension of the treatment period, demethylation variation patterns were the most significant under 200 nmol·mol^−1^ ozone stress (Wang et al., 2023). Those suggested that demethylation may play a vital role in plants responses to ozone stress. In addition, when not exposed to ozone treatment, the S1 generation (S1-X-0d) exhibited a lower overall methylation level, whereas the S1 plants continuously exposed to ozone stress displayed increased DNA methylation levels compared to the S1-X-0d, but still lower than the control (S1-CK) (**Figure 3**, **Table 1**). This indicates that the S1 generation may have inherited the epigenetic memory without exposure to ozone, and this epigenetic memory can be maintained under repeated ozone re-treatment conditions. We also found that millet may be more sensitive to ozone stress during the reproductive growth period compared to the vegetative growth period. Additionally, under ozone stress, DNA methylation variations between different generations of millet can exist at different sites, but mainly at the CNG sites, which is consistent with previous research findings (Wang et al., 2023).

Sustained environmental stress exhibits plasticity in epigenetic modifications, which can be transmitted through mitosis/meiosis, playing a key role in plant genome evolution and adaptation to adversity (Lucibelli et al., 2022; Cao and Chen, 2024; Yadav et al., 2024). Therefore, we further analyzed the inheritance modes of DNA methylation variations. There are six modes of inheritance for DNA methylation mutations, with the two most frequent being A-A-B-B (26.72%) and A-A-B-A (48.73%). Notably, the A-A-B-B, A-B-B-B, and A-B-C-C patterns, considered to show stress memories during ozone stress, accounted for 39.1% of total variation sites. Not only can types A-A-B-B and A-B-B-B be stably inherited to the next generation after long-term treatment in the S0 generation, but another type (A-B-C-C) also showed new methylation pattern variations, suggesting differences in epigenetic responses between vegetative and reproductive growth stages under ozone stress (Begcy and Dresselhaus, 2019). These epigenetic variations induced by ozone stress have an inheritance effect, making methylation variations a potential new mechanism for plants to adapt to ozone stress (Weinhold, 2018; Wang et al., 2023).

In the mutation band sequencing results, we successfully identified six potential candidate sequences (**Table 2**): a biological rhythm regulation protein (MS7), polyadenylate-binding proteins (MS54, MS178), a membrane-associated calcium-binding protein (MS92), an ABC transporter (MS120), and a histone acetyltransferase (MS192). Three genes (MS7, MS92, and MS178) had their methylation variation patterns stably preserved in the S1 generation (A-A-B-B). Research have showed that these functional proteins are involved in plant hormone signal transduction (JA), phosphorylation processes, and genome stability, indicating that these genes may play an important role in dealing with ozone stress (Kobae et al., 2006; Leshem et al., 2007; Shin et al., 2012). Although the methylation variation patterns of MS54, MS120, and MS192 were not stably preserved in the S1 generation (A-A-B-A), they still displayed a new variation pattern as they were inherited across generations. Previous research has found that the mentioned functional proteins (MS54, MS120) play an active role in processes such as photosynthetic pigment synthesis, stomatal opening, and the transport of stress-resistant substances (Martinoia et al., 2002; Leshem et al., 2007). Additionally, we discovered a gene for histone acetyltransferase (MS192) that actively participates in the dynamic regulation with histone deacetylases involved in plant vegetative growth, fruit ripening, and responses to both biotic and abiotic stresses (Kumar et al., 2021; Lin et al., 2022).

## 5 Conclusion

Crop productivity directly depends on growth and development and its adaptability to different environmental stresses. However, dynamic changes in DNA methylation play a crucial role in their response to abiotic stress. We found that, compared to the control group, ozone can induce hypomethylation and hypermethylation variations in the millet genome, with demethylation being more widespread. Furthermore, with the increase in treatment generations, the S1 generation is more prone to both demethylation and hypermethylation variations (at CNG sites). During the generational transmission process, we also identified the heritable methylation variation patterns (A-A-B-B, A-B-B-B, and A-B-C-C) and a new cumulative variation pattern that emerge with increasing treatment generations (A-A-A-B). Additionally, during ozone stress, three genes (MS7, MS92, and MS178) had their methylation variation patterns stably preserved in the S1 generation. Thus, such epigenetic changes in the millet genome could be an important regulatory mechanism for adapting to ozone or other environmental stresses. These findings provide additional genetic information and a theoretical basis for the molecular mechanisms of plant resistance to O_3_ stress.

## Data Availability

The original contributions presented in the study are included in the article/**Supplementary Material**, further inquiries can be directed to the corresponding author/s.
