# Supplementary material for: Ozone stress-induced DNA methylation variations and their transgenerational inheritance in foxtail millet
Source: Front Plant Sci. 2024 Sep 25;15:1463584. doi: 10.3389/fpls.2024.1463584 (PMC11461238; doi:10.3389/fpls.2024.1463584)
Supplement: Supplementary file 1 [file Table1.docx]

**Table S1.** Sequences of adapters and primers for MSAP analysis.

| **Primer Types** | **Adapters(Primers)** | **Sequences（5'-3'）** |
| --- | --- | --- |
| Adapter | EcoRⅠ-adapter Ⅰ | CTCGTAGACTGCGTACC |
|  | EcoRⅠ-adapter Ⅱ | AATTGGTACGCAGTC |
|  | H/M-adapter Ⅰ | GATCATGAGTCCTGCT |
|  | H/M-adapter Ⅱ | CGAGCAGGACTCATGA |
| Pre-amplification primer | EcoRⅠ + A | GACTGCGTACCAATTCA |
|  | H/M+ 0 | ATCATGAGTCCTGCTCGG |
| Selective-amplification primer | EcoRⅠ -AAC | GACTGCGTACCAATTCAAC |
|  | EcoRⅠ -AGA | GACTGCGTACCAATTCAGA |
|  | EcoRⅠ -AAG | GACTGCGTACCAATTCAAG |
|  | EcoRⅠ -ATC | GACTGCGTACCAATTCATC |
|  | EcoRⅠ -ACA | GACTGCGTACCAATTCACA |
|  | EcoRⅠ -AGG | GACTGCGTACCAATTCAGG |
|  | EcoRⅠ -ACT | GACTGCGTACCAATTCACT |
|  | EcoRⅠ -AGC | GACTGCGTACCAATTCAGC |
|  | EcoRⅠ -ACC | GACTGCGTACCAATTCACC |
|  | EcoRⅠ -ACG | GACTGCGTACCAATTCACG |
|  | H/M-TTA | ATCATGAGTCCTGCTCGGTTA |
|  | H/M-TTG | ATCATGAGTCCTGCTCGGTTG |
|  | H/M-TGA | ATCATGAGTCCTGCTCGGTGA |
|  | H/M-TTC | ATCATGAGTCCTGCTCGGTTC |
|  | H/M-TGT | ATCATGAGTCCTGCTCGGTGT |
|  | H/M-TCC | ATCATGAGTCCTGCTCGGTCC |
|  | H/M-TGC | ATCATGAGTCCTGCTCGGTGC |
|  | H/M-TCT | ATCATGAGTCCTGCTCGGTCT |
|  | H/M-TAC | ATCATGAGTCCTGCTCGGTAC |
|  | H/M-TCG | ATCATGAGTCCTGCTCGGTCG |
